# A rare case of concurrent prostatic acinar adenocarcinoma and primary prostatic urothelial carcinoma: case report and literature review

**DOI:** 10.3389/fmed.2026.1830418

**Published:** 2026-04-23

**Authors:** Yan Xia, Yamei Deng

**Affiliations:** 1 Cancer Center, Department of Pathology, Zhejiang Provincial People’s Hospital, Affiliated People’s Hospital, Hangzhou Medical College, Hangzhou, Zhejiang, China; 2 Department of Urology, Hangzhou Hospital of Traditional Chinese Medicine, Hangzhou, Zhejiang, China

**Keywords:** composite tumor, follow-up, primary prostatic urothelial carcinoma, prostatic acinar adenocarcinoma, radical prostatectomy, targeted therapy

## Abstract

**Background:**

Acinar adenocarcinoma is the most common subtype of prostate cancer, while primary prostatic urothelial carcinoma is clinically rare. The composite tumor with both occurring simultaneously within the prostate is even rarer, with limited clinical reports and diagnostic and therapeutic experience available to date.

**Case presentation:**

This article reports a case of a 75-year-old male with concurrent prostatic acinar adenocarcinoma and primary prostatic urothelial carcinoma. The patient was admitted to the hospital due to an elevated prostate-specific antigen (PSA) level detected during physical examination for 2 years. Preoperative magnetic resonance imaging (MRI) showed a nodule in the left peripheral zone of the prostate, and radical prostatectomy was performed subsequently. Postoperative pathological diagnosis revealed prostatic acinar adenocarcinoma (Gleason score 4 + 3, pT3b stage) and primary prostatic urothelial carcinoma *in situ* with microinvasion (<0.1 cm). The patient received disitamab vedotin targeted therapy 6 months after surgery, and multi-point biopsy of the bladder mucosa 10 months after surgery showed no tumor tissue. At the time of this report, the patient had undergone 15 months of follow-up with no evidence of tumor recurrence.

**Conclusion:**

The composite tumor of concurrent prostatic acinar adenocarcinoma and primary prostatic urothelial carcinoma is clinically rare. Definitive diagnosis requires comprehensive judgment based on imaging examinations, histopathological observation and immunohistochemical detection. Individualized treatment plans should be formulated based on multidisciplinary consultation, and long-term close follow-up of patients is necessary to monitor tumor recurrence.

## 1 Introduction

Prostate cancer is one of the most common malignant tumors of the male genitourinary system, with acinar adenocarcinoma accounting for 90 to 95% of cases and being the predominant pathological subtype. Primary prostatic urothelial carcinoma accounts for only 1 to 4% of prostatic malignant tumors (1), which is clinically rare and characterized by greater aggressiveness and poorer prognosis (2). Notably, secondary invasion of the prostate by bladder urothelial carcinoma is far more common than primary prostatic urothelial carcinoma. For this reason, rigorous assessment of the bladder—including cystoscopy, imaging, and histopathological biopsy—is mandatory to exclude secondary involvement before establishing a diagnosis of primary prostatic urothelial carcinoma (1, 3). Combined prostatic neoplasms with synchronous occurrence of acinar adenocarcinoma and primary urothelial carcinoma are extremely rare. Its pathogenesis remains unclear, and there is a lack of uniform standards for its diagnosis and treatment. Herein, we report a confirmed case of this rare neoplasm and review the relevant literature to investigate its pathological features, key points of differential diagnosis, individualized treatment strategies and prognostic factors, aiming to provide a clinical reference for the diagnosis and management of such diseases.

## 2 Case presentation

A 75-year-old male patient was admitted to our hospital due to progressively elevated serum prostate-specific antigen (PSA) over a two-year period, accompanied by increased urinary frequency, urgency, and nocturia for more than 1 month. He denied experiencing abdominal pain, distension, lower abdominal mass, fever, fatigue, or weight loss. His mental status, appetite, and sleep were satisfactory, without significant abnormalities in bowel movements, urination, or body weight.

**Figure 1:**
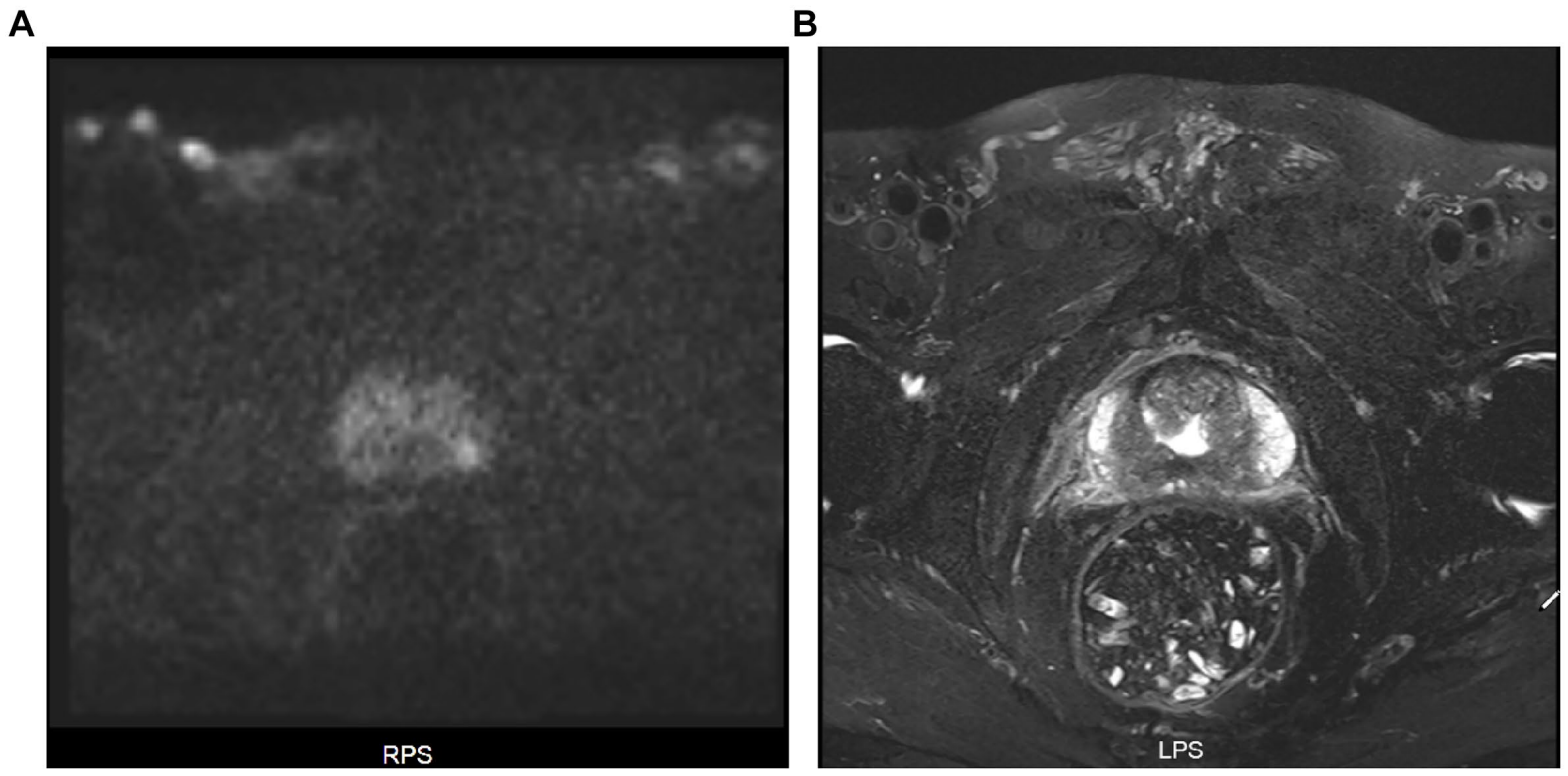
**(A)** Axial diffusion-weighted imaging (DWI) sequence of the prostate (phase encoding direction: RPS), where the hyperintense signal area indicates the prostate cancer lesion; **(B)** Axial T2-weighted enhanced magnetic resonance imaging (MRI) of the prostate (phase encoding direction: LPS), where the irregularly enhanced central area is the primary focus of prostate cancer.

Four years before the patient’s admission, contrast-enhanced computed tomography (CT) of the urinary tract revealed a soft tissue density shadow adjacent to the bladder neck. He subsequently underwent transurethral resection of the bladder, and postoperative pathology confirmed a diagnosis of benign prostatic hyperplasia (BPH). Two years ago, a routine examination detected an elevated PSA level, which continued to increase progressively thereafter. Six months prior, serum PSA testing showed a total PSA of 6.953 μg/L. On the same day, prostate contrast-enhanced MRI (Figure 1) demonstrated the following findings: irregular prostate contour; nodular abnormal signals in the transition zone (Prostate Imaging Reporting and Data System [PI-RADS] score 2, suggestive of benign hyperplasia); a 9 mm × 7 mm nodule in the left peripheral zone (PI-RADS score 4, biopsy recommended); and no abnormalities in the bilateral seminal vesicles, bladder, pelvic lymph nodes, or bony structures. Positron emission tomography/computed tomography (PET/CT) performed half a month prior to admission showed changes consistent with post-transurethral resection of bladder tumor; increased 18F-prostate-specific membrane antigen (18F-PSMA) uptake in a nodule of the left prostate lobe, suggestive of prostate cancer; and partial, mildly increased uptake in multiple bilateral inguinal lymph nodes, suggestive of an inflammatory process.

The patient was admitted for comprehensive evaluations to rule out surgical contraindications. Clinically, there was a high suspicion of prostate cancer. After detailed consultation, the patient and his family declined needle biopsy and opted to proceed directly to laparoscopic radical prostatectomy.

**Figure 2:**
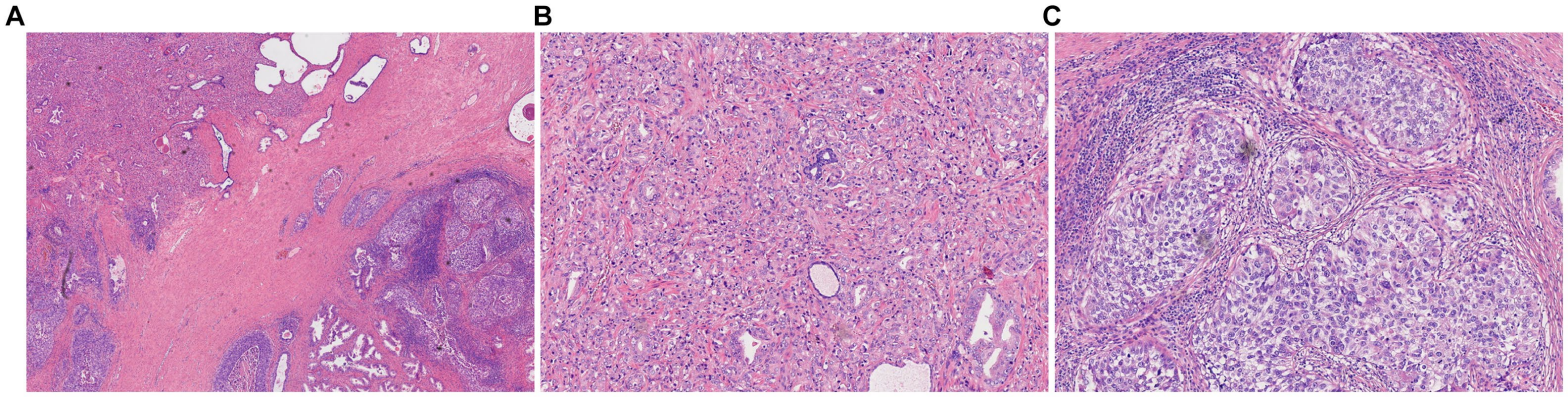
Histopathological features of prostatic collision tumor. **(A)** Low-power view (×40) showing acinar adenocarcinoma (upper left) and primary urothelial carcinoma (lower right) with clear demarcation. **(B)** Area of acinar adenocarcinoma, where tumor cells show cribriform and acinar structures (×100). **(C)** Area of primary urothelial carcinoma, where tumor cells are arranged in ductal and solid patterns (×100).

**Figure 3:**
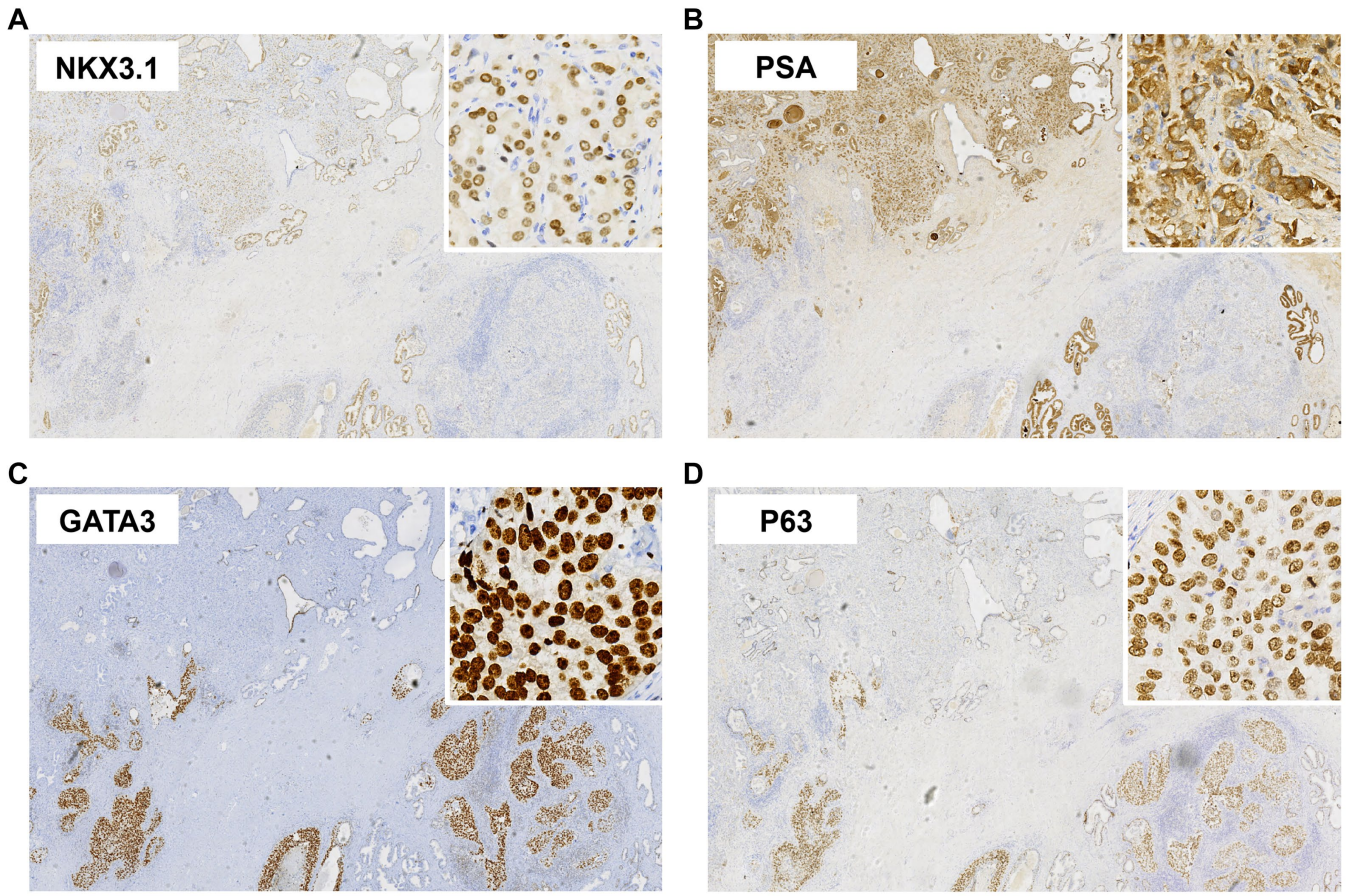
Immunohistochemical staining results of the prostatic collision tumor (original magnification ×20, with high-power insets at ×400). In the acinar adenocarcinoma component (upper left): **(A)** NKX3.1 shows nuclear positive expression, and **(B)** PSA shows cytoplasmic positive expression. In the primary urothelial carcinoma component (lower section): **(C)** GATA3 and **(D)** p63 show nuclear positive expression.

Pathological examination revealed that the prostate tumor consisted of two components (Figure 2): (1) Prostatic acinar adenocarcinoma with a Gleason score of 4 + 3 = 7 (World Health Organization/International Society of Urological Pathology [WHO/ISUP] grade 3/5), accounting for approximately 10% of the total lobular volume, invading the left seminal vesicle, and staged as pT3b; (2) Prostatic urothelial carcinoma *in situ* with localized microinvasion (<0.1 cm), constituting approximately 25% of the total lobular volume. Immunohistochemical findings were as follows (Figure 3): Prostatic acinar adenocarcinoma: NK3 homeobox 1 (NKX3.1)(+), cytokeratin 34βE12 (CK34βE12)(−), tumor protein p63 (p63)(−), cytokeratin 5/6 (CK5/6)(−), *α*-methylacyl-CoA racemase (AMACR, P504S)(+), prostate-specific antigen (PSA)(+), GATA binding protein 3 (GATA3)(−), cytokeratin 7 (CK7)(−), cytokeratin 20 (CK20)(focal+); Urothelial carcinoma: NKX3.1(−), CK34βE12(+), p63(+), CK5/6(+), AMACR, P504S(+), PSA(focal+), GATA3(+), CK7(+), CK20(+). Human epidermal growth factor receptor 2 (HER2), Programmed death-ligand 1 (PD-L1), and DNA mismatch repair (MMR) proteins were all examined in both tumor components. Results were as follows: HER2 immunoreactivity was scored 2 + in the urothelial carcinoma component, while the acinar adenocarcinoma component was negative for HER2. PD-L1 assessment yielded a combined positive score (CPS) of approximately 5, with positive staining predominantly localized to the urothelial carcinoma component. Both components retained intact expression of PMS2, MSH2, MSH6, and MLH1, consistent with a proficient mismatch repair (pMMR) phenotype.

Following multidisciplinary team (MDT) consultation, the patient received systemic therapy with disitamab vedotin via intravenous infusion starting at 6 months postoperatively. At 10 months after surgery, multiple bladder mucosal biopsies were obtained, which showed no histopathological evidence of tumor recurrence. During follow-up, PSA was measured every 3 months; pelvic MRI and PSMA PET/CT were performed every 6 months; and cystoscopy was conducted every 6 months to screen for urothelial recurrence. At the time of reporting, the patient remained free of tumor recurrence after 16 months of follow-up.

## 3 Discussion

Prostatic collision tumors composed of acinar adenocarcinoma and primary prostatic urothelial carcinoma are extremely rare, with only a small number of cases reported worldwide (4–7). The clinical presentation of such composite tumors lacks specificity, often leading to misdiagnosis as isolated prostate cancer or lower urinary tract disorders and subsequent delayed early diagnosis. Patients typically present with persistent or progressive elevation of serum PSA, which is associated with PSA secretion by the acinar adenocarcinoma component. Some patients may experience lower urinary tract symptoms, such as frequency, urgency, or dysuria, which are often attributed to urothelial carcinoma involving the prostatic urethra or concurrent BPH (6). Metastasis-related symptoms may occur in the advanced stage of the disease. Imaging evaluation requires multimodal integration and interpretation. PSMA PET/CT exhibits high sensitivity for the detection of acinar adenocarcinoma, enabling visualization of both primary and metastatic lesions, but demonstrates limited sensitivity for urothelial carcinoma (8). Contrast-enhanced prostate MRI has a significant advantage in detecting acinar adenocarcinoma, which typically presents as a peripheral zone lesion with low signal intensity on T2-weighted imaging (T2WI), high signal intensity on diffusion-weighted imaging (DWI), early contrast enhancement, and PI-RADS scores of 4–5 (9). Primary urothelial carcinoma typically manifests as an irregular lesion in the prostatic base or transition zone, showing mixed signal intensity on T2WI and heterogeneous contrast enhancement (10). However, when both components coexist, the lesion often presents as a single mass, making preoperative differentiation challenging and necessitating postoperative pathological examination for definitive confirmation (5).

In 2002, Mai et al. (11) first proposed that prostate cancer and urothelial carcinoma may coexist within the prostate gland, representing an extremely rare subtype of prostate cancer. Their study identified areas with urothelial carcinoma features within both the ductal components and the infiltrated tissues of the prostate tumor. These tumor areas were all high-grade, most of which exhibited ductal characteristics and often coexisted with adenocarcinoma areas showing papillary or sieve-like structures. Our present case demonstrated the concurrent presence of acinar adenocarcinoma with an acinar architecture and urothelial carcinoma with a tubular architecture. Unlike the report by Mai et al. (11), the urothelial carcinoma in our case was urothelial carcinoma *in situ* with minimal invasion, representing an early-stage malignancy. This finding further supports its primary origin in the prostate.

Notably, such prostatic collision tumors are clinically mostly presented as synchronous multiple primary cancers, and the distinction between synchronous and metachronous tumors is of great reference significance for clinical decision-making. Synchronous tumors are defined as two malignant tumors diagnosed simultaneously or successively within 6 months, whereas metachronous tumors refer to those with an interval of more than 6 months. Synchronous tumors are generally associated with higher aggressiveness and a greater risk of progression, whereas metachronous tumors usually show a relatively indolent clinical course (12).

Prostatic acinar adenocarcinoma exhibits typical histopathological features, primarily characterized by disrupted acinar architecture, cellular atypia, and stromal infiltration. Immunohistochemical staining reveals positive expression of NKX3.1, PSA, and P504S. Primary prostatic urothelial carcinoma arises from the urothelium of the prostatic urethra or the distal transitional epithelium of prostatic ducts (1). Its histopathology is similar to that of bladder urothelial carcinoma, presenting as tubular, solid, or *in situ* growth patterns with marked cytologic atypia, but with greater invasiveness. Positive immunohistochemical expression of GATA3, p63, CK(34βE12), and CK5/6 facilitates differentiation from acinar adenocarcinoma (3), a pattern consistently observed in published series (6, 7).

Such composite tumors require careful differentiation from the following conditions (3): (1) Prostatic ductal adenocarcinoma: It typically presents with papillary or sieve-like structures, shows diffuse positive immunohistochemical staining for PSA and NKX3.1, and is negative for urothelial markers—distinct from the present case; (2) Bladder urothelial carcinoma invading the prostate: It is typically associated with a history of primary bladder disease, with tumor infiltration predominantly in the urethral segment and transition zone. No bladder lesions were identified in this case, and both preoperative and postoperative bladder biopsies were negative, supporting the diagnosis of a primary tumor; (3) Metastatic urothelial carcinoma: It typically zprzesents with symptoms from the primary site and findings of upper urinary tract masses. This case lacks such evidence and can thus be excluded; (4) Acinar adenocarcinoma with urothelial metaplasia: The metaplastic tissue exhibits no significant dysplasia or mitotic figures and lacks malignant features. In the present case, the two tumor components express distinct markers for acinar adenocarcinoma and urothelial carcinoma, respectively, with no cross-expression. Combined with histopathological morphology, this enables a definitive diagnosis and rules out the possibility of transformation or metaplasia within a single tumor subtype. Additionally, unlike previous studies reporting PSA negativity (6, 7), focal positive expression was observed in the urothelial carcinoma *in situ* of this case. This finding may provide new insights into its pathogenesis.

The mechanisms underlying the concurrent occurrence of prostatic adenocarcinoma and urothelial carcinoma remain unclear, with several prevailing theories: (1) Both the bladder and the prostate originate from the urogenital ridge and share molecular similarities that may confer analogous genetic predispositions and potentially common carcinogenic pathways (12); (2) The androgen receptor plays a pivotal role in prostate cancer (13) and may also promote the initiation, progression, and proliferation of urothelial carcinoma (14); (3) The field cancerization theory proposes that under persistent exposure to adverse carcinogenic stimuli, both prostatic acinar cells and urothelial cells acquire and accumulate genetic alterations, ultimately transforming into malignant tumor cells (15).

For such synchronous tumors, comprehensive therapeutic strategies covering both histological types are required (12). Currently, there is no unified standard for the clinical management of primary prostatic urothelial carcinoma. It is widely acknowledged that early diagnosis and radical prostatectomy confer benefits to patients (16). According to the 2022 World Health Organization (WHO) Classification of Urinary and Male Genital Tumors, MMR protein testing is recommended to assess DNA mismatch repair function and guide immunotherapy eligibility (3). HER2 is a key therapeutic target in urothelial carcinoma. Disitamab vedotin is recommended for patients with HER2-positive advanced or metastatic urothelial carcinoma (17). The PD-L1 combined positive score (CPS) helps stratify patients who may benefit from immune checkpoint inhibitors; a low CPS indicates a limited likelihood of response to single-agent immunotherapy.

The present patient presented with concurrent pT3b prostatic acinar adenocarcinoma and primary urothelial carcinoma *in situ* with microinvasion. Urothelial carcinoma is more aggressive and carries a higher risk of bladder recurrence (1). After radical prostatectomy, the acinar adenocarcinoma was locally controlled. Immunohistochemical staining demonstrated intact DNA MMR function and low PD-L1 expression by the SP263 assay. Although HER2 2 + immunohistochemical status was noted without confirmatory ERBB2 gene amplification testing by fluorescence *in situ* hybridization (FISH), adjuvant disitamab vedotin was recommended following MDT discussion to target the HER2 positive urothelial component and reduce the risk of recurrence (17). The acinar adenocarcinoma component was monitored by serum PSA, and no immediate adjuvant endocrine therapy was administered due to the absence of residual lesions. At 10 months postoperatively, multiple-site bladder mucosal biopsies revealed no evidence of tumor. For urothelial carcinoma, long-term monitoring via cystoscopy and imaging studies is essential. If recurrence is detected during follow-up, radical cystectomy may be considered.

Similar to previous literature reports, synchronous prostatic acinar adenocarcinoma and primary prostatic urothelial carcinoma are more common in elderly males, with clinical manifestations mainly including elevated PSA and lower urinary tract symptoms. All cases show high consistency in pathological morphology and immunophenotype, and most patients have a favorable short-term prognosis after radical prostatectomy (6, 7). The prognosis of prostatic acinar adenocarcinoma combined with primary prostatic urothelial carcinoma depends on the staging and grading of both tumor components, with significant individual variability. In the present case, as the urothelial carcinoma was still at an early stage, the patient remained recurrence-free at 16 months postoperatively following active treatment and close follow-up. The distinctive feature of this case is that the urothelial carcinoma component was characterized by *in situ* carcinoma with microinvasion and was treated with adjuvant disitamab vedotin, which can provide an important reference for the clinical management of similar rare cases.

## 4 Conclusion

Cases of concurrent prostatic acinar adenocarcinoma and primary prostatic urothelial carcinoma are rare, with their pathogenesis remaining unclear. Currently, there is no unified standard for their clinical management. Based on a definitive pathological diagnosis, further studies involving larger sample sizes and longer follow-up durations are still required to provide evidence for optimizing diagnostic and therapeutic strategies.

## Data Availability

The original contributions presented in the study are included in the article/supplementary material, further inquiries can be directed to the corresponding author.
